# *Trypanosoma cruzi* iron superoxide dismutases: insights from phylogenetics to chemotherapeutic target assessment

**DOI:** 10.1186/s13071-022-05319-2

**Published:** 2022-06-06

**Authors:** Jéssica Hickson, Lucas Felipe Almeida Athayde, Thainá Godinho Miranda, Policarpo Ademar Sales Junior, Anderson Coqueiro dos Santos, Lúcia Maria da Cunha Galvão, Antônia Cláudia Jácome da Câmara, Daniella Castanheira Bartholomeu, Rita de Cássia Moreira de Souza, Silvane Maria Fonseca Murta, Laila Alves Nahum

**Affiliations:** 1 René Rachou Institute, Oswaldo Cruz Foundation (Functional genomics of parasites group; Biosystems informatics, bioengineering and genomic group), Belo Horizonte, Minas Gerais Brazil; 2 Department of Genetics, Ecology and Evolution, Institute of Biological Sciences, Federal University of Minas Gerais, Belo Horizonte, Brazil; 3 Department of Clinical and Toxicological Analysis, Federal University of Rio Grande do Norte State, Natal, Rio Grande do Norte Brazil; 4 Promove College of Technology, Belo Horizonte, Minas Gerais Brazil

**Keywords:** *Trypanosoma cruzi*, Iron superoxide dismutase, Antioxidant defense, Phylogenetic inference, Molecular evolution, Drug target

## Abstract

**Background:**

Components of the antioxidant defense system in* Trypanosoma cruzi* are potential targets for new drug development. Superoxide dismutases (SODs) constitute key components of antioxidant defense systems, removing excess superoxide anions by converting them into oxygen and hydrogen peroxide. The main goal of the present study was to investigate the genes coding for iron superoxide dismutase (FeSOD) in *T. cruzi* strains from an evolutionary perspective.

**Methods:**

In this study, molecular biology methods and phylogenetic studies were combined with drug assays. The FeSOD-A and FeSOD-B genes of 35 *T. cruzi* strains, belonging to six discrete typing units (Tcl–TcVI), from different hosts and geographical regions were amplified by PCR and sequenced using the Sanger method. Evolutionary trees were reconstructed based on Bayesian inference and maximum likelihood methods. Drugs that potentially interacted with *T. cruzi* FeSODs were identified and tested against the parasites.

**Results:**

Our results suggest that *T. cruzi* FeSOD types are members of distinct families. Gene copies of FeSOD-A (*n* = 2), FeSOD-B (*n* = 4) and FeSOD-C (*n* = 4) were identified in the genome of the *T. cruzi* reference clone CL Brener. Phylogenetic inference supported the presence of two functional variants of each FeSOD type across the *T. cruzi* strains. Phylogenetic trees revealed a monophyletic group of FeSOD genes of *T. cruzi* TcIV strains in both distinct genes. Altogether, our results support the hypothesis that gene duplication followed by divergence shaped the evolution of *T. cruzi* FeSODs. Two drugs, mangafodipir and polaprezinc, that potentially interact with *T. cruzi* FeSODs were identified and tested in vitro against amastigotes and trypomastigotes: mangafodipir had a low trypanocidal effect and polaprezinc was inactive.

**Conclusions:**

Our study contributes to a better understanding of the molecular biodiversity of *T. cruzi* FeSODs. Herein we provide a successful approach to the study of gene/protein families as potential drug targets.

**Graphical Abstract:**

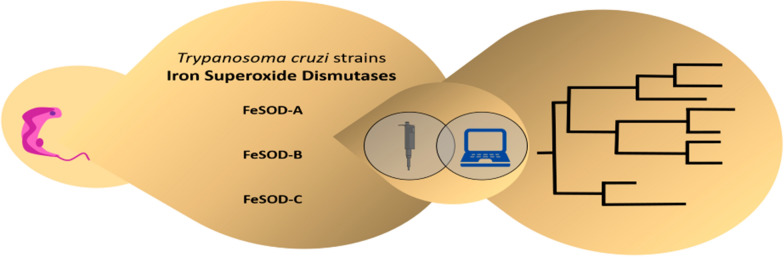

**Supplementary Information:**

The online version contains supplementary material available at 10.1186/s13071-022-05319-2.

## Background

The protozoan parasite *Trypanosoma cruzi* (Kinetoplastida: Trypanosomatidae) is the causative agent of Chagas disease. This zoonosis occurs mainly in Latin America, but it is estimated that more than six million individuals are infected worldwide [1]. The underreporting of infection and death rates represents an unprecedented challenge. Based on genetic diversity, *T. cruzi* is currently classified into six discrete typing units (DTUs: TcI–TcVI) [2, 3]. A seventh DTU, named TcBat, is a specific genotype that infects bats [4]. Different *T. cruzi* strains and clones display extensive morphological, biological, immunological, biochemical and pharmacological differences, which directly interfere in the clinical state of individuals with Chagas disease.

The current therapy for Chagas disease is limited to the drugs benznidazole (BZ) and nifurtimox (NFX) [5], which are very toxic, and the efficacy of treatment remains low in the chronic phase of the disease. Other factors influencing the treatment efficacy of these drugs in terms of cure include the treatment duration, drug dose, age of patient, geographical origin and individual patient’s immune system. In addition, the occurrence of naturally resistant *T. cruzi* strains may be one of the most important variables in the failure to cure infected populations [5–8].

Trypanosomatids are subjected to intense oxidative stress caused by exposure to toxic subproducts, such as nitric oxide (^•^NO), peroxynitrite (ONOO^−^/ONOOH), superoxide anion (O_2_^•−^), hydrogen peroxide (H_2_O_2_) and hydroperoxides (containing hydroperoxide functional group ROOH), that are derived from cellular metabolism and from external agents (e.g. drug metabolites) and host immune mediators. However, trypanosomatids have an important and unique mechanism for the trypanothione-dependent detoxification of peroxides that differs from the mechanism found in vertebrates and, as such, is indicated as a rational target for chemotherapy [9]. Many enzymes involved in antioxidant defense are distributed in diverse cellular compartments, which are activated against various oxidants. Antioxidant contents of *T. cruzi* are a determinant of the parasite survival or death at the moment of the infection [10].

Superoxide dismutases (SODs) constitute an essential defense element against oxidative damage in various organisms [11–13]. These metalloproteases (EC 1.15.1.1) remove excess O_2_^•−^ by converting them into O_2_ and H_2_O_2_ [14]. They are classified according to their prosthetic group (copper and zinc, iron or manganese, or nickel) and are found in different cellular localizations. Eukaryotes usually have copper-zinc (Cu/ZnSODs) and manganese (MnSODs) enzymes [15]. Iron SODs (FeSODs) are found in prokaryotes, protozoans, plants and algae [16]. As FeSOD is absent in the human host, this enzyme could be considered a potential target for chemotherapy against trypanosomatids [17, 18]. Some studies of *T. cruzi* FeSODs used sequence and structural data to characterize them biochemically [19, 20], while others focused on gene expression, drug association [21–23], functional studies [24, 25], infectivity and virulence [26, 27] and phylogenetic approaches [16, 28]. Taken together, these studies highlight the presence of distinct FeSODs acting in different cellular compartments (cytosol, glycosome and mitochondria). The vital roles of FeSODs also make them potential targets for new treatment or medicine, repositioning strategies against Chagas disease [17].

Phylogenetics may be applied to study the evolutionary history of gene and protein families and to access their molecular biodiversity [29–35]. This approach also provides a framework for functional prediction of molecular targets of interest [16, 29, 33]. The identification of gene/protein family members may reveal structural and/or functional variants, which is an essential aspect towards inferring the evolutionary history of potential drug targets.

In the present study, we investigated the molecular biodiversity and evolutionary relationships of FeSOD-A and FeSOD-B among different *T. cruzi* strains. We also evaluated the trypanocidal effect of two drugs (mangafodipir and polaprezinc) that potentially interact with *T. cruzi* FeSODs.

## Methods

### *Trypanosoma cruzi* strains

Thirty-five *T. cruzi* strains isolated from human patients, domestic vectors and sylvatic reservoirs or vectors, from different geographic areas, were used in this study (see Table 1; Additional file 1: Figure S1). Samples were obtained from the *T. cruzi* cryobanks at the René Rachou Institute-FIOCRUZ (Professor Zigman Brener Collection), Federal University of Minas Gerais-UFMG (Professor Egler Chiari Collection) and the Oswaldo Cruz Institute-FIOCRUZ (Collection of *Trypanosoma* from Wild and Domestic Mammals and Vectors-COLTRYP). All *T. cruzi* strains used in the present study had been classified previously according to six DTUs (Tcl–TcVI), as described elsewhere [2].

**Table 1:** Description of the *Trypanosoma cruzi* strains used in this study

Discrete typing unit	Strain	Origin	Host	Sus^a^	Reference for Sus
TcI	2137	Rio Grande do Norte	*Homo sapiens*	ND	–
	2549	Rio Grande do Norte	*Homo sapiens*	ND	–
	Colombiana	Colombia^b^	*Homo sapiens*	R	[37]
	Quaraizinho	Rio Grande do Sul	*Triatoma infestans*	S	[37]
	RS–21	Rio Grande do Sul	*Panstrongylus megistus*	S	[39]
	SC28	Santa Catarina	*Didelphis marsupialis*	R	[37]
TcII	Berenice	Minas Gerais	*Homo sapiens*	S	[37]
	Ernane	Goiás	*Homo sapiens*	S	[37]
	Gilmar	Minas Gerais	*Homo sapiens*	S	[37]
	MR	Rio Grande do Sul	*Triatoma infestans*	S	[37]
	São Felipe	Bahia	*Triatoma infestans*	S	[37]
	VL–10	Minas Gerais	*Homo sapiens*	R	[37]
	Y	São Paulo	*Homo sapiens*	MR	[37]
TcIII	C00113	Goiás	*Monodelphis domestica*	ND	–
	C00370	Pará	*Rhodnius pictipes*	ND	–
	C00566	Mato Grosso do Sul	*Dasypus novemcinctus*	ND	–
	PEBA18	Rio Grande do Norte	*Euphractus sexcinctus*	ND	–
	PL0213	Rio Grande do Norte	*Panstrongylus lutzi*	ND	–
	RN19	Rio Grande do Norte	*Panstrongylus lutzi*	ND	–
	SM76	Rio Grande do Norte	*Homo sapiens*	ND	–
TcIV	AM64	Amazonas	*Homo sapiens*	MR	[40]
	C00041	Mato Grosso do Sul	*Thrichomys pachyurus*	ND	–
	C00471	Mato Grosso do Sul	*Oecomys mamorae*	ND	–
	C00524	Mato Grosso do Sul	*Triatoma* spp*.*	ND	–
	C00526	Mato Grosso do Sul	*Triatoma* spp*.*	ND	–
	C00601	Espírito Santo	*Triatoma vitticeps*	ND	–
TcV	3253	Rio Grande do Sul	*Homo sapiens*	ND	–
	Bug2149cl10	Rio Grande do Sul	*Triatoma infestans*	R	[74]
	JM	Minas Gerais	*Homo sapiens*	S	[37]
TcVI	Buriti	Rio Grande do Sul	*Triatoma infestans*	S	[37]
	CL	Rio Grande do Sul	*Triatoma infestans*	S	[37]
	CL Brener clone	Rio Grande do Sul	*Triatoma infestans*	S	[37]
	FL	Rio Grande do Sul	*Triatoma infestans*	S	[37]
	Luna	Argentina^c^	*Homo sapiens*	S	[37]
	RS–12	Rio Grande do Sul	*Homo sapiens*	ND	–

*MR* Medium resistance, *ND* not determined,* R* resistant, *S* susceptible

^a^Sus: in vivo drug susceptibility to benznidazole (BZ) and/or nifurtimox (NFX), as previously described [37, 39]

^b^*T. cruzi* strain isolated from Colombia

^c^*T. cruzi* strain isolated from Argentina

Epimastigotes of *T. cruzi* strains were maintained in liquid liver infusion tryptose medium at 28 °C [36]. Genomic DNA extraction from *T. cruzi* strains and subsequent electrophoresis of DNA fragments were carried out as previously described [21]. The in vivo susceptibility to BZ and NFX of some *T. cruzi* strains have been previously characterized [37–40] (Table 1).

### Identification of potential homologs

Searching for potential homologs of molecular targets is the first step in determining whether the molecular targets belong to a gene/protein family. The presence of multiple potential homologs in the genome and predicted proteome of the different *T. cruzi* strains indicates that the target of the study belongs to a family. In this context, the *T. cruzi* proteomes were searched using the Pfam 34.0 identifiers to identify FeSOD potential homologs in UniProt, as reported previously [41]. Potential FeSOD homologs encoded in the genome of different *T. cruzi* strains were identified in TriTrypDB release 53 [30] using the Pfam [42] identifiers PF00081 and PF02777. *Trypanosoma cruzi* strains with more than one gene imply that FeSODs are members of a multigenic family.

### FeSOD gene copy number

Analysis of the copy number of the genes encoding FeSOD-A and FeSOD-B was performed with all nucleotide sequences used in our phylogenetic reconstruction and the *T. cruzi* FeSOD-C gene sequence retrieved from the TriTrypDB (Additional file 2: Figure S2). Sequence similarity search against the *T. cruzi* reference CL Brener genome assembly was performed using reads obtained by two systems: the PacBio system [43] and the Illumina HiSeq system [44]. These genome data have not been published yet (DC Bartholomeu, personal communication).

The complete open reading frame of each gene was identified and translated into its corresponding amino acid sequence, and checked for the presence of internal stop codons. The predicted FeSOD genes, annotated to each distinct gene, were obtained according to the matches found between our gene sequences and the new genome assembly. To confirm the annotated genes, we evaluated the read depth in the corresponding regions on the assembly. The short reads were mapped using the BWA-MEM algorithm [45]. For each genome, depth was measured by SAMtools [46] with mapping quality 30, and the depth and coverage of the FeSOD gene regions were calculated. The copy number of each FeSOD in the genome assembly was obtained according to the ratio between gene and genome depth.

### Gene amplification and sequencing

FeSOD genes from the 35 *T. cruzi* strains, corresponding to the six DTUs TcI–TcVI, were sequenced (Table 1). Primers used to amplify the CDS sequence of the FeSOD-A and FeSOD-B genes are listed in Additional file 3: Table S1. FeSOD primers were designed based on the conserved nucleotide sequences of *T. cruzi* sequences shown in Table 2. All PCR amplifications were carried out using the Platinum® PCR SuperMix (Invitrogen, Thermo Fisher Scientific, Waltham, MA, USA), according to the manufacturer’s protocol. PCR products of 637 bp (FeSOD-A) and 588 bp (FeSOD-B) from genomic DNA (Additional file 3: Figure S3) were separated by 1% agarose gel electrophoresis. Amplicons with the expected sizes were purified using the QIAquick® PCR purification Kit (Qiagen, Hilden, Germany). After the presence of FeSODs in the *T. cruzi* strains was confirmed, the purified PCR products were directly used for Sanger sequencing [47]. Each PCR amplification and sequencing were performed five times (2 technical and 3 biological replicates). For the internal primers, the procedure was performed twice (2 replicates). The Phred-Phrap-Consed package [48] was used for sequence assembly and processing.

**Table 2:** Description of sequences in the datasets used for phylogenetic reconstruction

Species	Strain	Isoform^a^	UniProt identifiers	European Nucleotide Archive (ENA) identifiers	Reference Sequence (RefSeq) identifiers	Protein Data Bank (PDB) identifiers	Reference	Length (nt)	Sus^b^
*T. cruzi*	CL Brener clone	FeSOD-A	Q4DCQ3	EAN90306.1	XM_807064	4DVH	[23]	702	S
						4H3E	[75]		
*T. cruzi*	Tulahuen clone 2	FeSOD-A	O02615	AAC47548.2	NA	NA	[19]	636	S
*T. cruzi*	Y clone 4	FeSOD-A	Q2TJ60	AAX84933.1	NA	NA	[21]	637	S
	Y clone 16	FeSOD-A	Q2TJ60	AAX84936.1	NA	NA	[21]	637	R
*T. cruzi*	17LER	FeSOD-A	Q2TJ65	AAX84930.1A	NA	NA	[21]	637	R
	17WTS	FeSOD-A	Q2TJ65	AX84931.1	NA	NA	[21]	637	S
	Barra Seca	FeSOD-A	Q2TJ65	AAX84932.1A	NA	NA	[21]	637	S
	YUYU	FeSOD-A	Q2TJ65	AX84938.1	NA	NA	[21]	637	R
*T. cruzi*	CL Brener clone	FeSOD-A	Q2TJ61	AAX84934.1A	NA	NA	[21]	637	S
	JA	FeSOD-A	Q2TJ61	AX84935.1AA	NA	NA	[21]	637	S
	São Felipe	FeSOD-A	Q2TJ61	X84937.1AAX	NA	NA	[21]	637	S
	VL-10	FeSOD-A	Q2TJ61	84939.1	NA	NA	[21]	637	R
*T. cruzi*	CL Brener clone	FeSOD-B	Q4DI29	EAN92179.1	XM_808937	2GPC	[76]	588	S
							[23]		
*T. cruzi*	Tulahuen clone 2	FeSOD-B	O02616	AAC47549.1	NA	NA	[19]	588	S

*MR* Medium resistant, *NA* not applicable, *R* resistant, *S* susceptible

3D structure not available in PDB

^a^FeSOD-A,-B: Iron superoxide dismutase isoform A and B, respectively

^b^Sus: in vivo drug susceptibility to BZ and/or NFX, as previously described [37, 39]

### Multiple sequence alignments

Two datasets containing potential FeSOD homologs were selected for analysis. These datasets include nucleotide sequences from *T. cruzi* obtained in the present study and *T. cruzi* FeSOD nucleotide sequences retrieved from public databases, such as the European Nucleotide Archive (ENA 2021) [49] and the Reference Sequence Database (RefSeq release 205) [50] (Table 2). Each FeSOD isoform (FeSOD-A and FeSOD-B) was analyzed separately. Nucleotide sequences of each dataset were aligned using MUSCLE [51] with default parameters as implemented in MEGA X package [52]. Multiple sequence alignments were manually edited and gaps were excluded to increase data quality. Conserved, variable and parsimony informative sites were identified in each alignment by using MEGA X. These sites were accessed to check the phylogenetic signal of each alignment. We applied Kimura 2-parameter (FeSOD-A dataset) and HKY85 (FeSOD-B dataset) as the best-fit models, as indicated by jModelTest 2.1.10 [53]. For the two alignments, we estimated the proportion of invariable sites.

### Phylogenetic reconstruction

Edited sequence alignments were used for phylogenetic reconstruction by applying two character-based methods. For the maximum likelihood method implemented in PhyML [54], bootstrap values were obtained from 1000 pseudoreplicates. For the Bayesian inference implemented in MrBayes 3.2.7 [55], a variant of the Markov chain Monte Carlo (MCMC) method was used. MCMC analyses were run as four chains (1 cold and 3e heated) for 10,000,000 generations and sampled every 100 generations. Of the initial samples, 25% were discarded as “burn-in.” Support values for Bayesian inference were estimated as Bayesian posterior probabilities. The average standard deviation of split frequencies (ASDSF) and the potential scale reduction factor (PSRF) were evaluated in Bayesian trees. ASDSF < 0.01 suggests that the two independent sessions become increasingly similar trees. A PSRF of approximately 1 indicates that the generation of trees converged. Additionally, an estimated sample size (ESS) > 100 ensures that the parameters adopted are not subsampled. Evolutionary trees were rooted using the midpoint method and edited in FigTree 1.4.4 [56].

### Identification of potential drugs interacting with FeSOD

DrugBank is a unique bioinformatics and chemoinformatics database that combines chemical and pharmacological drug properties with sequence and structural information associated with potential target pathways [57]. This database contains drugs with experimental data (mass spectrometry and nuclear magnetic resonance), drugs in phases I/II/III of investigation and drugs approved by the US Food and Drug Administration (FDA), Health Canada, European Medicines Agency (EMA) and other national agencies. All nucleotide sequences included in the present study were translated into protein sequences and used in the DrugBank 5.1.4 search tool to identify drugs that potentially interact with *T. cruzi* FeSODs. Those drugs identified as potentially interacting with *T. cruzi* FeSODs were tested in vitro against amastigotes and trypomastigotes based on toxicity profiles and pharmacological properties.

### Evaluation of the in vitro anti-*T. cruzi* activity of selected drugs, cellular toxicity and selectivity

The in vitro anti-*T. cruzi* activity was evaluated on L929 cells (mouse fibroblasts) infected with the Tulahuen strain of the parasite expressing the *Escherichia coli* β-galactosidase as reporter gene, according to the method described previously [58]. This assay allows evaluation of anti-*T. cruzi* activity of drugs against both the amastigote and trypomastigote forms of the parasite.

Polaprezinc, a zinc-related medicine, and mangafodipir, a contrast agent used in magnetic resonance imaging (both from MedChemExpress [MCE], Monmouth Junction, NJ, USA), were tested at concentrations ranging from 62.5 to 1000 μM, for an incubation period of 96 h. Each dilution was tested in triplicate. The controls were uninfected cells, untreated infected cells and infected cells treated with benznidazole at 1 μg/ml (3.8 μM, positive control) or DMSO (1%, v/v). The results were expressed as the percentage of *T. cruzi* growth inhibition in drug-tested cells as compared to the infected cells and untreated cells, and IC_50_ values (concentration that inhibits 50% of the growth of the parasites) were calculated by linear interpolation. Active drugs were evaluated for cytotoxicity and selectivity on uninfected fibroblasts [58]. The results were expressed as the difference in the reduction percentage among treated and untreated cells, and the CC_50_ determined (drug concentration that inhibits 50% of the L929 cell viability). The selectivity index (SI) was calculated as the ratio of the CC_50_ value in the L929 cells to the IC_50_ value of *T. cruzi* cells.

## Results

### Identification of potential FeSOD family members

FeSODs have a conserved domain architecture according to Pfam. Most *T. cruzi* FeSOD enzymes have one N-terminal (PF00081) and one C-terminal (PF02777) domain. To identify FeSOD family members in *T. cruzi*, we used the respective Pfam identifiers to search the UniProt (proteome data) and TriTrypDB (genomic data) databases and found that the number of potential gene/protein homologs identified varied across different *T. cruzi* strains.

Pfam identifiers were used to search for the potential FeSOD homologs in the proteome of the *T. cruzi* CL-Brener (UniProt: UP000002296) [59], Dm28c (UP000246121) [60] and TCC (UP000246078) [60] strains available in the UniProt database (February 2021). The search for the two Pfam domains (PF00081 and PF02777) in the *T. cruzi* FeSOD sequences resulted in the recovery of a total of 9, 7 and 11 proteins in the predicted proteomes, respectively. The search for *T. cruzi* FeSOD sequences in the TriTrypDB database for these domains showed 13, 9 and 14 sequences in the three genomes, respectively (Table 3). No sequence analyzed in the present sudy has only the PF00081 domain. On the other hand, searching only for the PF02777 domain showed four, two and two genes in the three genomes, respectively. These results and the number of the genes encoding FeSODs in the different strains (Table 3) suggest the existence of *T. cruzi* FeSODs with only the PF02777 domain and that *T. cruzi* FeSODs are members of a gene/protein family. Altogether, these results may highlight processes shaping the evolution of *T. cruzi* FeSODs.

**Table 3:** *T. cruzi* FeSOD sequences containing the Pfam domains PF00081 and/or PF02777

*T. cruzi* strain	Only the PF00081 domain	Only the PF02777 domain	Both the PF00081 and PF02777 domains	Number of sequences analyzed
Brazil A4	0	2	5	7
CL-Brener Clone Esmeraldo-like/Non-Esmeraldo-like	0	4	9	13
Dm28c 2014	0	1	2	3
Dm28c 2017	0	2	7	9
Dm28c 2018	0	2	7	9
Marinkellei strain B7	0	2	2	4
Sylvio X10/1-2012	0	1	4	5
TCC	0	2	12	14
YC6	0	1	8	9

Pfam domains: PF00081 (alpha-hairpin domain) and/or PF02777 (C-terminal domain) as identified in the TriTrypDB database

### Estimating FeSOD gene copy number

The *T. cruzi* CL-Brener reference genome sequence currently available at the TriTrypDB database is fragmented and contains some inaccuracies [59]. To better estimate the complete repertoire of FeSOD genes in *T. cruzi* CL-Brener, we searched for these sequences in a PacBio- and Illumina-based assembly. This analysis revealed the presence of two FeSOD-A and four FeSOD-B genes. Sequences from these two distinct genes differ primarily by the presence of an extension at the 5ʹ end in the FeSOD-A gene, which is absent in the FeSOD-B gene. The FeSOD-A gene correspondences are located on two different scaffolds (TcBrS006 and TcBrS020) with high similarity to each other. On the other hand, the FeSOD-B gene correspondences were found on four different scaffolds (TcBrS024, TcBrS074, TcBrS110 and TcBrS188). In TcBrS024 and TcBrS110, we observed genes with high similarity and longer length at the 3’ end as compared to the TcBrS074 and TcBrS188 sequences, which are also highly similar to one another. With respect to FeSOD-C, two gene correspondences were found on scaffold TcBrS091 and two other correspondences on scaffold TcBrS112. Analysis of read depth and coverage confirmed the predicted annotated FeSOD genes of each distinct gene (GenBank: MZ825448-MZ825457). We detected complete coverage (100%) in all cases. The mean and normalized depth (gene/genome depth) were represented here (Additional file 3: Table S2). We confirmed the presence of two copies of FeSOD-A genes, four copies of the FeSOD-B and four copies of the FeSOD-C genes.

### FeSOD multiple sequence alignments

The FeSOD-A and FeSOD-B genes of 35 *T. cruzi* strains (GenBank: OL620009-OL620078) were amplified by PCR and sequenced as described in the Methods section. All *T. cruzi* strains presented one amplicon for the FeSOD-A gene (approx. 637 bp) and one amplicon for the FeSOD-B gene (approx. 588 bp). Sequence assembly data confirmed that most of the total gene length for each FeSOD type was recovered (Additional file 3: Table S3).

Two datasets of nucleotide sequences were aligned (Additional file 4: Figure S4) and submitted to phylogenetic inference analysis: dataset I (46 T*. cruzi* FeSOD-A sequences with 573 sites) and dataset II (36 T*. cruzi* FeSOD-B sequences with 484 sites). The Tulahuen genes (FeSOD-A and FeSOD-B) were removed because they seemed to create tree artifacts. Analyses of these two datasets revealed the nucleotide diversity of FeSOD genes across the different *T. cruzi* strains analyzed here.

The alignment between the FeSOD-A and FeSOD-B genes revealed a high conservation of nucleotide sequences of each distinct gene. However, despite the sequence conservation, each dataset had enough phylogenetic signal to be used in the tree reconstruction. The best-fit model for each sequence alignment was estimated by jModelTest 2.1.10 [53]. The Kimura 2-parameter was estimated for dataset I and HKY85 was estimated for dataset II. The proportion of invariable sites was estimated.

A comparison by similarity among the sequences used in phylogenetic reconstruction (FeSOD-A and FeSOD-B) and FeSOD-C genes (used to determine the copy number in *T. cruzi* CL Brener genome) is shown in Additional file 5: Figure S5. The FeSOD-C sequences TcBrA4_0028220, TcCLB.511737.3 and Tc_MARK_2024 were not inserted into the alignment because they do not include the sequence code of the PF00081 domain. Information on all of these sequences is available in Additional file 6: Table S4.

### Phylogeny of *T. cruzi* FeSOD genes

Bayesian- and maximum likelihood-based phylogenies were reconstructed with sequences obtained in the present study and other *T. cruzi* FeSOD sequences retrieved from public databases. Both methods retrieved similar tree topologies for the two datasets analyzed here (Additional file 7: Figure S6).

**Fig. 1:**
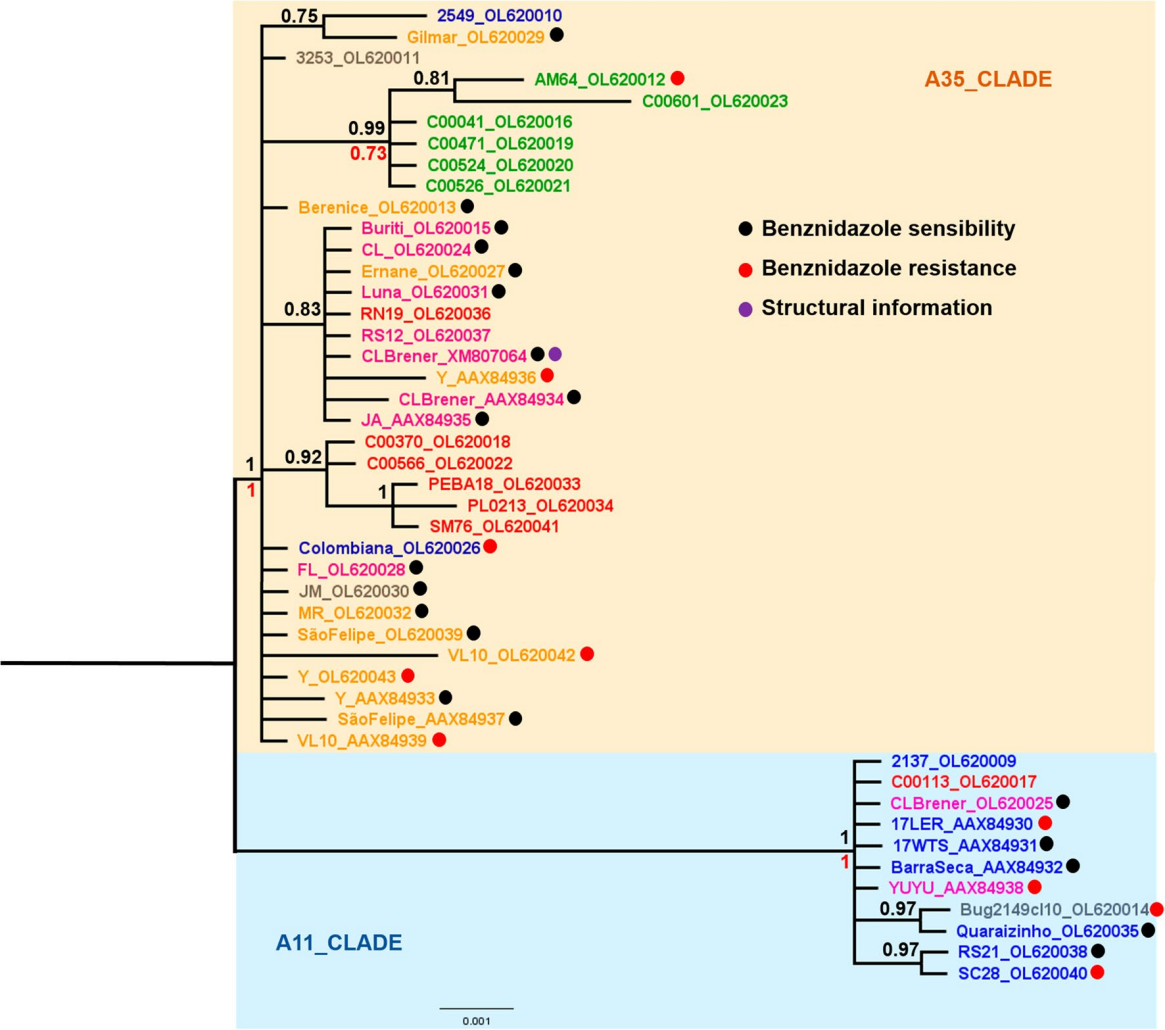
Evolutionary relationships of 46 *Trypanosoma cruzi* FeSOD-A gene sequences. Nucleotide sequences obtained in this study are named according to the *T. cruzi* strains (see Table 1) and identifiers come from GenBank. Sequences from public databases are named according to the ENA database, except for CL_Brener.XM_807064, which comes from the RefSeq database (see Table 2). The Tulahuen gene sequence was removed because it appears to cause a tree artifact. Discrete typing units are highlighted: TcI (blue), TcII (orange), TcIII (red), TcIV (green), TcV (gray) and TcVI (pink). The alignment comprises a total of 573 sites. The phylogeny was reconstructed by two methods using the best fit model (Kimura-2 parameter) and estimation of the proportion of invariable sites. In the Bayesian inference, support values for each node were estimated as posterior probability (numbers in black above node). In the maximum likelihood analysis, they were estimated using the bootstrap method (numbers in red below node). Only support values higher > 70% are shown

**Fig. 2:**
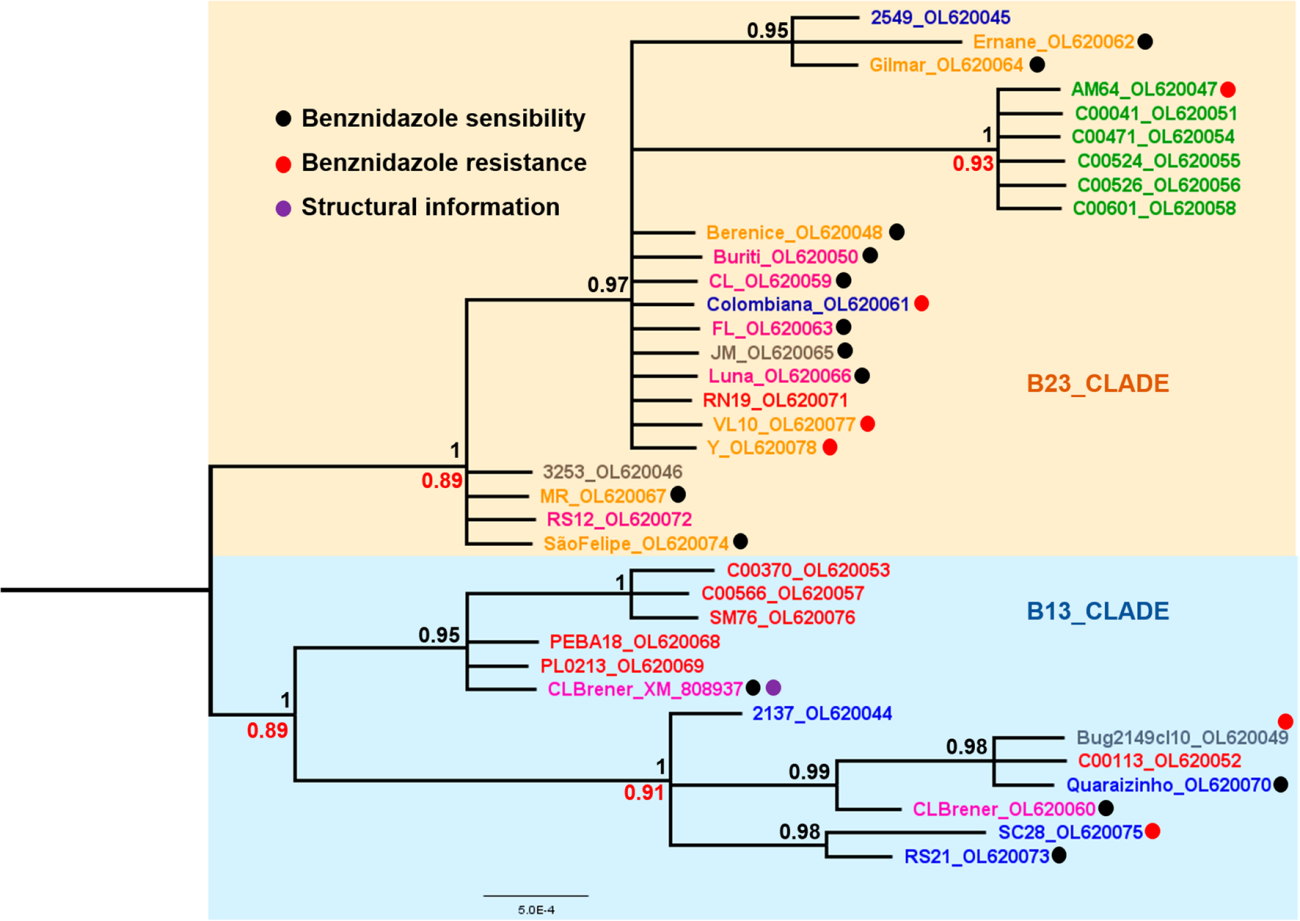
Evolutionary relationships of 36 T*. cruzi* FeSOD-B gene sequences. Nucleotide sequences obtained in this study are named according to the *T. cruzi* strains (see Table 1) and identifiers come from GenBank. Sequences from public databases are named according to the strain. Identifiers come from the RefSeq database (CL_Brener.XM_808937) (see Table 2). The Tulahuen gene sequence was removed because it appears to cause a tree artifact. Discrete typing units are highlighted: TcI (blue), TcII (orange), TcIII (red), TcIV (green), TcV (gray) and TcVI (pink). The alignment comprises a total of 484 sites. The phylogeny was reconstructed by two methods using the best fit model (HKY85) and estimation of the proportion of invariable sites. In the Bayesian inference, support values for each node were estimated as posterior probability (number in black above node). In the maximum likelihood analysis, they were estimated using the bootstrap method (numbers in red below node). Only support values >70% are shown

The phylogenetic tree of the *T. cruzi* FeSOD-A sequences have two main clades (A35_CLADE and A11_CLADE) with high statistical support values that may represent two gene subtypes (Fig. 1). Similar results were obtained for the *T. cruzi* FeSOD-B gene tree (Fig. 2) with two main clades (B23_CLADE and B13_CLADE) with high statistical support values. The latter results suggest for the first time the existence of two functional subtypes of the FeSOD-B gene.

The average standard deviation of all split frequencies and the potential scale reduction factor of the FeSOD-A gene tree were 0.009862 and 1.001, respectively. For the FeSOD-B gene tree, these values were 0.009909 and 1.001. To optimize the PSRF, we sampled 10,000,000 generation every 100 generations. A PSFR close to 1 indicates that generations have converged. When the estimated sample size is > 100, the indication is that the parameters have not been subsampled.

We did not observe congruence between the parasite host and geographic location with the evolutionary relationships presented in each phylogeny. Evolutionary relationships of the *T. cruzi* FeSOD gene families (FeSOD-A and FeSOD-B) show a monophyletic group formed by genes of the TcIV *T. cruzi* strains. The common ancestor among these genes may reflect the natural history of *T. cruzi* (Figs. 1, 2).

In summary, the FeSOD-B gene tree shows better statistical support by both phylogenetic methods than the FeSOD-A gene tree. Therefore, the evolutionary relationships among FeSOD-B genes are better resolved compared to those of FeSOD-A genes. Gene family trees show relationships among genes and not taxa (Figs. 1, 2).

Tree annotations are based on experimental evidence of some sequences described elsewhere. Experimentally characterized genes can be used to predict some functional features of uncharacterized ones present in the same clade (Figs. 1, 2).

### Selection of drugs interacting with FeSOD

Amino acid sequences from FeSOD-A and FeSOD-B genes sequenced in the present study and those retrieved from the UniProt database were used to search DrugBank for chemical drugs that can interact with these sequences. Two drugs that potentially interact with *T. cruzi* FeSODs were identified and tested in vitro against amastigotes and trypomastigotes as described in the Methods section. Mangafodipir had a low trypanocidal effect and polaprezinc was inactive against the parasite.

Polaprezinc is a chelated form of zinc and L-carnosine that shows therapeutic activity for the treatment of pressure ulcers and other intestinal lesions [61, 62], is used in cancer chemotherapy [63, 64], presents therapeutic effects on cardiac function [65] and has a protective effect against respiratory diseases [66].

Mangafodipir is a manganese chelate responsible for releasing free manganese ions into the blood. This drug is used as a contrast agent in diagnostics [67]. It showed promising results as a cytoprotectant during the treatment of heart diseases and neuropathies [67].

### In vitro trypanocidal activity of drugs interacting with FeSOD

Potential FeSOD interacting drugs were assayed against amastigotes and trypomastigotes of the *T. cruzi* Tulahuen strain. Polaprezinc at concentrations of 1000, 500, 250 and 125 μM caused cell death of fibroblasts (Additional file 8: Table S5). At a concentration of 62.5 μM it showed only 14% trypanocidal activity. Thus, this drug was considered to be inactive against *T. cruzi.* Mangafodipir caused a reduction in the parasite population, but the trypanocidal effect (69%) occurred at the highest concentration (1000 μM), with a high IC_50_ value of 839 μM. In addition, it was also cytotoxic against mouse fibroblasts L929 cells with CC_50_ value of 2298 μM. Thus, this drug was not approved for in vivo testing because it exhibits a low selectivity towards parasites (SI: 2.7) (Additional file 8: Table S5).

## Discussion

The antioxidant defense system in trypanosomatids is composed of many enzymes that act in concert against various oxidants. FeSODs are important enzymes in this system. In the present study, we investigated the molecular biodiversity and evolutionary relationships of FeSODs in different *T. cruzi* strains, including searches of different public databases. Our results suggest that *T. cruzi* FeSODs are members of gene/protein superfamilies.

Knowledge of multigene family members improves understanding of the origin and evolution of genes and gene products. Moreover, such knowledge may provide information for the functional prediction of uncharacterized genes and, subseqently, for the development of therapeutic strategies involving these genes. Homologous genes and proteins may perform identical, similar, or complementary functions. Therefore, it is important that drug candidates interact with all members of multigene families to ensure the trypanocidal effect.

Regarding the number of copies of FeSODs genes in the *T. cruzi* CL-Brener genome, our results show two copies of the FeSOD-A gene, four copies of the FeSOD-B gene, with two similar pairs, and four copies of the FeSOD-C gene. These results agree with those of a previous analysis that showed the presence of two FeSOD-A and four FeSOD-B gene copies in *T. cruzi* Tulahuen clone 2 [19]. Two copies of FeSOD-A were also found in the 17WTS strain [21]. In the present study, the amino acid sequences encoded by the FeSOD-A and FeSOD-B genes in the *T. cruzi* CL-Brener were found to share 69% similarity.

We constructed a diagram showing the differences among the FeSOD-A, -B and -C genes. For building the diagram, one sequence of each FeSOD type was chosen. The selection was done based on the sequences that contained more information in the literature (sequences with greater reliability). We also added information obtained for the predicted gene copy number in the *T. cruzi* CL-Brener genome: two copies (FeSOD-A) or four copies (FeSOD-B and FeSOD-C). In general, information available in the literature and public databases is scarce. In this context, our work provides a better understanding of these proteins in *T. cruzi* (Additional file 8: Figure S7).

In order to reconstruct the phylogenetic history of FeSODs using gene sequences of different *T. cruzi* strains, we investigated two different isoforms (FeSOD-A and FeSOD-B) present in this parasite. The main difference found between the FeSOD-A and FeSOD-B genes is at the 5’ end, where a portion composed of 15 amino acids is present in the former and absent in the latter. This N-terminal extension depicts an overall pattern of a mitochondrial signal peptide [19]. Our datasets also reveal a high similarity among the FeSOD-A and FeSOD-B genes separately. Our phylogenetic inferences for the *T. cruzi* FeSOD-A and FeSOD-B genes are represented by trees with two main clades which suggest the existence of two subtypes of each FeSOD type (Figs. 1, 2). This diverse enzymatic profile in different cell locations ensures a more effective action against oxidants [20, 68, 69]. Dufernez et al. [16] reconstructed the evolutionary history among amino acid sequences of FeSODs from *Trypanosoma brucei* and other organisms. Their phylogenetic relationship suggests that FeSODs of subtypes B1 and B2 emerged independently from specific ancestors by gene duplication in each species of the database (*T. brucei*, *T. congolense*, *T. cruzi, T. vivax*, and *Leishmania* species). In *Leishmania* species, types B1 and B2 are recovered in separate clades, while in *Trypanosoma* species these proteins are brought together in a single clade. While such topology may be explained as the result of gene duplications, which occurred independently in *Trypanosoma* species and strains, such an evolutionary scenario is unlikely.

Dufernez et al. [16] suggest the existence of a correlation among FeSODs. These authors propose the occurrence of more than one lateral gene transfer event that gave rise to multiple FeSODs in *T. brucei* [16]. Based on the results of the present study, we also propose that the two possible functional variants of FeSOD-A and FeSOD-B originated by duplication events followed by divergence.

We did not identify congruences by geographic region and host isolation of *T. cruzi* strains in the FeSOD gene trees (Figs. 1, 2). In contrast, we observed a clade well supported by statistical values, with a monophyletic group composed of all FeSOD-A and FeSOD-B genes from the TcIV *T. cruzi* strains in each phylogeny. It has been proposed [70] proposed the origin of the TcIV *T. cruzi* DTU group is due intraspecific hybridization events between DTUs TcI and TcII, generating the ancestral ecotypes of DTUs TcIII and TcIV. The *T. cruzi* TcIV strains contain several specific characteristics: (i) they are predominantly present in the Amazon region, where they mainly infect non-human primates; (ii) they exhibit a high virulence within a short pre-patent period in infected mice, producing wide tissue-tropism toward skeletal muscle, high parasitemia and mortality rates in the acute phase of infection [71].

We believe that these specific characteristics may reflect the common ancestry among FeSODs of TcIV *T. cruzi* strains. FeSODs are essential for the parasite to survive and for the infectious processes, suggesting that the evolution of these enzymes is aligned with the evolution of *T. cruzi*. More specifically, we propose that the set of evolutionary mechanisms which shaped the evolution of genes encoding FeSODs in the TcIV strains are plesiomorphic, as the ancestor that originated these *T. cruzi* strains diverged. In this context, our results corroborate those of previous studies using a multilocus sequence typing (MLST) scheme for *T. cruzi* genetic typing [28]. Using this approach, the authors analyzed 10 housekeeping genes, including FeSOD-A and FeSOD-B genes. These sequences are from 32 different *T. cruzi* strains belonging to six DTUs, and the phylogenetic tree displays every DTU as a monophyletic group [28]. It was only possible to obtain a monophyletic group of the TcIV *T. cruzi* strains when using four loci, including fragments of the FeSOD-B genes [28]. Together, these complementary results show that genotypic differences in FeSOD genes can define the phylogenetic signal that classifies TcIV *T. cruzi* strains.

Our dataset includes sequences that were used in studies that show important FeSOD roles in *T. cruzi* antioxidant defense against reactive oxygen and nitrogen species. FeSODB protects *T. cruzi* against peroxynitrite toxicity inside the phagosome by preventing its formation or by its reacting directly with the oxidant [69]. Previous studies showed that FeSODs favor the proliferation, survival and virulence of *T. cruzi*. [26, 27].

In the present study, we did not observe any correlation between the drug-resistant and drug-susceptible phenotype of *T. cruzi* strains analyzed in relation to the different clades of the phylogenetic trees. In the FeSOD-A and FeSOD-B trees, well-supported clades contain strains/clones showing different susceptibilities to BZ. Other studies investigating the expression and specific enzyme activity developed by our group have highlighted the strong indications that FeSODs are associated with the drug resistance mechanism used by *T. cruzi* [21, 39].

In this study, our search of the DrugBank database resulted in the identification of two drugs, mangafodipir and polaprezinc, that potentially interact with *T. cruzi* FeSODs. The in vitro activity of both drugs against trypomastigote and amastigote *T. cruzi* forms was evaluated. Polaprezinc was inactive against *T. cruzi*, and mangafodipir exhibited a low trypanocidal effect and low selectivity against *T. cruzi*. Although the drugs tested in vitro were not promising, the high conservation observed among the *T. cruzi* FeSOD gene sequences (Additional file 4: Figure S4) and the absence of FeSODs in humans that have manganese (MnSOD) or copper and zinc (Cu-ZnSODs) as a prosthetic group indicate the high potential of this enzyme as a target of new drugs. Literature data have shown that benzo[*g*]phthalazine and phthalazine derivatives are active against *T. cruzi* and show selective inhibitory effects on *T. cruzi* FeSOD enzyme activity in comparison with human CuZn-SOD [72, 73].

## Conclusions

Our phylogenetic inference study suggests the existence of two functional variants of each FeSOD analyzed across *T. cruzi* strains. We believe that these variants have been differentiating through duplication events followed by divergence over evolutionary time. This hypothesis is exclusive and well supported by other studies that indicate that the parasite needs several enzymes to provide efficient antioxidant protection in different cellular compartments. FeSOD genes of TcIV *T. cruzi* strains studied here belong to a monophyletic group, suggesting that the phylogenetic history of this DTU reflects the evolution of FeSODs. In future studies, we intend to reconstruct the phylogenetic history of other members of the *T. cruzi* antioxidant defense system. Additionally, we want perform in vitro testing against the parasite with other drugs that interact with these proteins, with the aim to find a new therapeutic agent against *T. cruzi*.

## Supplementary Information

**Additional file 1:** **Figure S1.** Nucleotide sequences (FeSOD-A and FeSOD-B genes) of the datasets used in this study.

**Additional file 2:** **Figure S2.** Alignment of sequences of potential *T. cruzi* FeSOD-C.

**Additional file 3**: **Table S1. **Primers to amplify *T. cruzi* FeSOD-A and FeSOD-B genes. Abbreviations: Temp, Predicted annealing temperature. **Figure S3.** PCR amplification products of FeSOD-A and FeSOD-B genes of 8 *T. cruzi* strains. Electrophoresis was performed in a 1% agarose gel stained with GelRed™ (Biotium). The molecular weight markers 1-kb Plus DNA Ladder (Invitrogen) were used. Abbreviations: NC, Negative control of reaction without template. ** Table S2.** Data of read depth and coverage analysis. The depth read values and coverage proportion in the genomic regions (GenBank: MZ825448–MZ825457) for each FeSOD type of genes and the respective normalized value (gene/genome depth). Depth was calculated using the value for each position of the gene. The coverage corresponds to the proportion of position that has reads in the gene, and normalized values are the depth of the genes divided by the mean of genome depth.** Table S3. **Sequence assembly information. Reads refers to the number of reads used for sequence assembly; Consensus refers to the length (nt) of the consensus sequence. Sequence assembly was performed using Phred-Phrap-Consed.

**Additional file 4**: **Figure S4.** Nucleotide sequence alignment of the datasets used in this study. Alignments were performed in MUSCLE as implemented in MEGA and edited by visual inspection. Different types of sites identified in each alignment are indicated in Table S1. Each edited alignment was further used for phylogenetic reconstruction.

**Additional file 5:** **Figure S5.** Alignment of sequences of the trees reconstructed in this study (FeSOD-A and FeSOD-B sequences), Tulahuen gene sequences (FeSOD-A and FeSOD-B sequences) and FeSOD-C genes (used to determine the copy number in the *T. cruzi* CL-Brener genome).

**Additional file 6:** **Table S4. **Information of sequences of the trees reconstructed in this study (FeSOD-A and FeSOD-B sequences) and FeSOD-C genes (used to determine the copy number in the *T. cruzi* CL-Brener genome).

**Additional file 7:** **Figure S6.** Trees of the datasets used in this study. Trees were reconstructed in MrBayes and PhyML.

**Additional file 8**: **Table S5.**In vitro trypanocidal activity, cytotoxicity and selectivity index of selected drugs. Superscripts: ^1^Reduction (%) of the amastigote and trypomastigote by drug; ^2^drug concentration that inhibits 50% of the growth of the amastigotes and trypomastigotes of *T*. *cruz*;^3^drug concentration that inhibits 50% of the L929 cell viability;^4^CC_50_ L929/IC_50_ *T*. *cruz*. IC_50_ and CC_50_ values were calculated by linear interpolation. The determination of CC_50_ of polaprezinc was not possible due low solubility in concentrations > 50 μM. Abbreviations: ND, Not determined. **Fig. S7.** The diagram illustrates FeSOD-A, FeSOD-B and FeSOD-C characteristics, including cellular localization (orange boxes), protein length (blue boxes), gene copies (green boxes) and protein domains (pink boxes). Information was retrieved from public databases and tools: ENA, Pfam, TriTrypDB, and UniProt. Reference sequences were selected for FeSOD-A (AAC47548.2), FeSOD-B (EAN92179.1) and FeSOD-C (TcCLB.511735.60). Only gene copy numbers were obtained by our prediction using the *T*. *cruzi* CL-Brener genome.

## Data Availability

Datasets used in the present study are available in the additional files. Sequences were deposited in GenBank (MZ825448-MZ825457) and (OL620009-OL620078).

## References

[1] WHO. Chagas disease (American trypanosomiasis). 2020. https://www.who.int/health-topics/chagas-disease. Accessed 27 Jul 2020.

[2] Zingales B, Andrade SG, Briones MR, Campbell DA, Chiari E, Fernandes O (2009). A new consensus for *Trypanosoma cruzi* intraspecific nomenclature: second revision meeting recommends TcI to TcVI. Mem Inst Oswaldo Cruz.

[3] Zingales B, Miles MA, Campbell DA, Tibayrenc M, Macedo AM, Teixeira MM (2012). The revised *Trypanosoma cruzi* subspecific nomenclature: rationale, epidemiological relevance and research applications. Infect Genet Evol.

[4] Hamilton PB, Lewis MD, Cruickshank C, Gaunt MW, Yeo M, Llewellyn MS (2011). Identification and lineage genotyping of South American trypanosomes using fluorescent fragment length barcoding. Infect Genet Evol.

[5] Sales Junior PA, Molina I, Fonseca SMM, Sánchez-Montalvá A, Salvador F, Corrêa-Oliveira R (2017). Experimental and clinical treatment of Chagas disease: a review. Am J Trop Med Hyg.

[6] Cançado JR (1999). Criteria of Chagas disease cure. Mem Inst Oswaldo Cruz.

[7] Sguassero Y, Cuesta CB, Roberts KN, Hicks E, Comandé D, Ciapponi A (2015). Course of chronic *Trypanosoma cruzi* infection after treatment based on parasitological and serological tests: a systematic review of follow-up studies. PLoS ONE.

[8] Molina I, Salvador F, Montalvá AS, Treviño B, Serre N, Avilés AS (2015). Toxic profile of benznidazole in patients with chronic Chagas disease: risk factors and comparison of the product from two different manufacturers. Antimicrob Agents Chemother.

[9] Santi AMM, Silva PA, Santos IFM, Murta SMF (2021). Downregulation of FeSOD-A expression in *Leishmania infantum* alters trivalent antimony and miltefosine susceptibility. Parasit Vectors.

[10] Ferrer-Sueta G, Radi R (2009). Chemical biology of peroxynitrite: kinetics, diffusion, and radicals. ACS Chem Biol.

[11] Plewes KA, Barr SD, Gedamu L (2003). Iron superoxide dismutases targeted to the glycosomes of *Leishmania chagasi* are important for survival. Infect Immun.

[12] McCord JM, Fridovich I (1969). Supeoxide dismutase: an enzymic function for erythrocuprein (hemocuprein). J Biol Chem.

[13] Neto VG, Ribeiro PR, Del-Bem LE, Bernal DT, Lima ST, Ligterink W (2018). Characterization of the superoxide dismutase gene family in seeds of two *Ricinus communis* L. genotypes submitted to germination under water restriction conditions. Environ Exp Bot.

[14] Fridovich I (1989). Superoxide dismutases. J Biol Chem.

[15] Sturtz LA, Diekert K, Jensen LT, Lill R, Culotta VC (2001). A fraction of yeast Cu, Zn-superoxide dismutase and its metallochaperone, CCS, localize to the intermembrane space of mitochondria. J Biol Chem.

[16] Dufernez F, Yernaux C, Gerbod D, Noel C, Chauveneta M, Wintjensd R (2006). The presence of four iron-containing superoxide dismutase isozymes in Trypanosomatidae: characterization, subcellular localization, and phylogenetic origin in *Trypanosoma brucei*. Free Radic Biol Med.

[17] Turrens JF (2004). Oxidative stress and antioxidant defenses: a target for the treatment of diseases caused by parasitic protozoa. Mol Aspects Med.

[18] Wilkinson SR, Prathalingam SR, Taylor MC, Ahmed A, Horn D, Kelly JM (2006). Functional characterization of the iron superoxide dismutase gene repertoire in *Trypanosoma brucei*. Free Radic Biol Med.

[19] Ismail SO, Paramchuk W, Skeiky YAW, Reed SG, Bhatia A, Gedamu L (1997). Molecular cloning and characterization of two iron superoxide dismutase cDNAs from *Trypanosoma cruzi*. Mol Biochem Parasitol.

[20] Villagrán ME, Marín C, Rodríguez-Gonzalez I, de Diego JA, Sánchez-Moreno M (2005). Use of an iron superoxide dismutase excreted by *Trypanosoma cruzi* in the diagnosis of Chagas disease: seroprevalence in rural zones of the state of Queretaro Mexico. Am J Trop Med Hyg.

[21] Nogueira FB, Krieger MA, Nirdé P, Goldenberg S, Romanha AJ, Murta SM (2006). Increased expression of iron-containing superoxide dismutase-A (TcFeSOD-A) enzyme in *Trypanosoma cruzi* population with *in vitro*-induced resistance to benznidazole. Acta Trop.

[22] Sanz AM, Gómez-Contreras F, Navarro P, Sánchez-Moreno M, Boutaleb-Charki S, Campuzano J (2008). Efficient inhibition of iron superoxide dismutase and of *Trypanosoma cruzi* growth by benzo[*g*]phthalazine derivatives functionalized with one or two imidazole rings. J Med Chem.

[23] Martínez A, Peluffo G, Petruk AA, Hugo M, Piñeyro D, Demicheli V (2014). Structural and molecular basis of the peroxynitrite-mediated nitration and inactivation of *Trypanosoma cruzi* iron-superoxide dismutases (Fe-SODs) A and B. J Biol Chem.

[24] Mateo H, Marín C, Pérez-Cordón G, Sánchez-Moreno M (2008). Purification and biochemical characterization of four iron superoxide dismutases in *Trypanosoma cruzi*. Mem Inst Oswaldo Cruz.

[25] Fracasso M, da Silva AD, Bottari NB, Monteiro SG, Garzon LR, de Souza LAF (2021). Resveratrol impacts in oxidative stress in liver during *Trypanosoma cruzi* infection. Microb Pathog.

[26] Piacenza L, Irigóin F, Alvarez MN, Peluffo G, Taylor MC, Kelly JM (2007). Mitochondrial superoxide radicals mediate programmed cell death in *Trypanosoma cruzi*: cytoprotective action of mitochondrial iron superoxide dismutase overexpression. Biochem J.

[27] Estrada D, Specker G, Martínez A, Dias PP, Hissa B, Andrade LO (2018). Cardiomyocyte diffusible redox mediators control *Trypanosoma cruzi* infection: role of parasite mitochondrial iron superoxide dismutase. Biochem J.

[28] Lauthier JJ, Tomasini N, Barnabé C, Rumi MMM, D’Amato AMA, Ragone PG (2012). Candidate targets for multilocus sequence typing of *Trypanosoma cruzi*: validation using parasite stocks from the Chaco region and a set of reference strains. Infect Genet Evol.

[29] Nahum LA, Pereira SL, Smolinski TG, Milanova MG, Hassanien AE (2008). Phylogenomics, protein family evolution, and the tree of life: an integrated approach between molecular evolution and computational intelligence. Studies in computational intelligence (SCI).

[30] Aslett M, Aurrecoechea C, Berriman M, Brestelli J, Brunk BP, Carrington M (2010). TryTripDB: a functional genomic resource for the trypanosomatidae. Nucleic Acids Res.

[31] Cuesta-Astroz Y, Scholte LLS, Pais FS, Oliveira G, Nahum LA (2014). Evolutionary analysis of the cystatin family in three *Schistosoma* species. Front Genet.

[32] Valdivia HO, Scholte LL, Oliveira G, Gabaldón T, Bartholomeu DC (2015). The *Leishmania* metaphylome: a comprehensive survey of *Leishmania* protein phylogenetic relationships. BMC Genomics.

[33] Mitchell JB (2017). Enzyme function and its evolution. Curr Opin Struc Biol.

[34] Scholte LLS, Mourão MM, Pais FS, Melesina J, Robaa D, Volpini AC (2017). Evolutionary relationships among protein lysine deacetylases of parasites causing neglected diseases. Infect Genet Evol.

[35] Anisimova M, Liberles DA, Philippe H, Provan J, Pupko T, von Haeseler A (2013). State-of the art methodologies dictate new standards for phylogenetic analysis. BMC Evol Biol.

[36] Camargo EP (1964). Growth and differentiation in *Trypanosoma cruzi*. I. origin of metacyclic trypanosomes in liquid media. Rev Inst Med Trop São Paulo.

[37] Filardi LS, Brener Z (1987). Susceptibility and natural resistance of *Trypanosoma cruzi* strains to drugs used clinically in Chagas disease. Trans R Soc Trop Med Hyg.

[38] Toledo MJO, Bahia MT, Carneiro CM, Martins-Filho OA, Tibayrenc M, Barnabé C (2003). Chemotherapy with benznidazole and itraconazole for mice infected with different *Trypanosoma cruzi* clonal genotypes. Antimicrob Agents Chemother.

[39] Murta SMF, Gazzinelli RT, Brener Z, Romanha AJ (1998). Molecular characterization of susceptible and naturally resistant strains of *Trypanosoma cruzi* to benznidazole and nifurtimox. Mol Biochem Parasitol.

[40] Teston APM, Monteiro WM, Reis D, Bossolani GDP, Gomes ML, de Araújo SM (2013). In vivo susceptibility to benznidazole of *Trypanosoma cruzi* strains from the western Brazilian Amazon. Trop Med Int Health.

[41] The UniProt Consortium (2018). UniProt: the universal protein knowledgebase. Nucleic Acids Res.

[42] Finn RD, Tate J, Mistry J, Coggill PC, Sammut SJ, Hotz HR (2008). The Pfam protein families database. Nucleic Acids Res.

[43] Wei Z, Zhang S (2018). NPBSS: a new PacBio sequencing simulator for generating the continuous long reads with an empirical model. BMC Bioinformatics.

[44] Liu L, Hu N, Wang B, Chen M, Wang J, Tian Z (2011). A brief utilization report on the Illumina HiSeq 2000 sequencer. Mycology.

[45] Li H (2013). Aligning sequence reads, clone sequences and assembly contigs with BWA-MEM. arXiv.

[46] Li H, Handsaker B, Wysoker A, Fennell T, Ruan J, Homer N (2009). The sequence alignment/map format and SAMtools. Bioinformatics.

[47] Sanger F, Nicklen S, Coulson AR (1977). DNA sequencing with chain-terminating inhibitors. Proc Natl Acad Sci USA.

[48] Machado M, Magalhães WCS, Sene A, Araújo B, Faria-Campos AC, Chanock SJ (2011). Phred-Phrap package to analyses tools: a pipeline to facilitate population genetics re-sequencing studies. Investig Genet.

[49] Amid C, Alako BTF, Kadhirvelu VB, Burdett T, Burgin J, Fan J (2020). The European nucleotide archive. Nucleic Acids Res.

[50] O’Leary NA, Wright MW, Brister JR, Ciufo S, Haddad D, McVeigh R (2016). Reference sequence (RefSeq) database at NCBI: current status, taxonomic expansion, and functional annotation. Nucleic Acids Res.

[51] Edgar RC (2004). MUSCLE: a multiple sequence alignment method with reduced time and space complexity. BMC Bioinformatics.

[52] Kumar S, Stecher G, Li M, Knyaz C, Tamura K (2018). MEGA X: molecular evolutionary genetics analysis across computing platforms. Mol Biol Evol.

[53] Darriba D, Taboada GL, Doallo R, Posada D (2012). jModelTest 2: more models, new heuristics and high-performance computing. Nat Methods.

[54] Guindon S, Dufayard J, Lefort V, Anisimova M, Hordijk W, Gascuel O (2010). New algorithms and methods to estimate maximum-likelihood phylogenies: assessing the performance of PhyML 3.0. Syst Biol.

[55] Ronquist F, Teslenko M, van der Mark P, Ayres DL, Darling A, Hohna S (2012). MrBayes 3.2: efficient Bayesian phylogenetic inference and model choice across a large model space. Syst Biol.

[56] FigTree. http://tree.bio.ed.ac.uk/software/figtree/. Accessed 20 Feb 2020.

[57] Wishart DS, Feunang YD, Guo AC, Lo EJ, Marcu A, Grant JR (2018). DrugBank 5.0: a major update to the DrugBank database for 2018. Nucleic Acids Res.

[58] Romanha AJ, Castro SL, Soeiro MNC, Lannes-Vieira J, Ribeiro I, Talvani A (2010). In vitro and in vivo experimental models for drug screening and development for Chagas disease. Mem Inst Oswaldo Cruz.

[59] El-Sayed NM, Myler PJ, Bartholomeu DC, Nilsson D, Aggarwal G, Tran A (2005). The genome sequence of *Trypanosoma cruzi*, etiologic agent of Chagas disease. Science.

[60] Berná L, Rodriguez M, Chiribao ML, Parodi-Talice A, Pita S, Rijo G (2018). Expanding an expanded genome: long-read sequencing of *Trypanosoma cruzi*. Microb Genom.

[61] Thomsen M, Vitetta L (2019). Zinc deficits, mucositis, and mucosal macrophage perturbation: is there a relationship?. Curr Opin Clin Nutr Metab Care.

[62] Liu Z, Xie W, Li M, Liu J, Liang X, Li T (2019). Intrarectally administered polaprezinc attenuates the development of dextran sodium sulfate-induced ulcerative colitis in mice. Exp Ther Med.

[63] Ye J, Zhang Z, Zhu L, Lu M, Li Y, Zhou J (2017). Polaprezinc inhibits liver fibrosis and proliferation in hepatocellular carcinoma. Mol Med Rep.

[64] Fujii H, Hirose C, Ishihara M, Iihara H, Imai H, Tanaka Y (2018). Improvement of dysgeusia by polaprezinc, a zinc-L-carnosine, in outpatients receiving cancer chemotherapy. Anticancer Res.

[65] Yoshikawa F, Nakajima T, Hanada M, Hirata K, Masuyama T, Aikawa R (2019). Beneficial effect of polaprezinc on cardiac function post-myocardial infarction: a prospective and randomized clinical trial. Medicine (Baltimore).

[66] Kimura K, Nakano Y, Sugizaki T, Shimoda M, Kobayashi N, Kawahara M (2019). Protective effect of polaprezinc on cadmium-induced injury of lung epithelium. Metallomics.

[67] Karlsson JOG, Andersson RGG, Jynge P (2017). Mangafodipir a selective cytoprotectant—with special reference to oxaliplatin and its association to chemotherapy-induced peripheral neuropathy (CIPN). Transl Oncol.

[68] Piacenza L, Peluffo G, Alvarez MN, Martínez A, Radi R (2013). *Trypanosoma cruzi* antioxidant enzymes as virulence factors in Chagas disease. Antioxid Redox Signal..

[69] Martínez A, Prolo C, Estrada D, Rios N, Alvarez MN, Piñeyro MD (2019). Cytosolic Fe-superoxide dismutase safeguards *Trypanosoma cruzi* from macrophage-derived superoxide radical. Proc Natl Acad Sci USA.

[70] Lewis MD, Llewellyn MS, Yeo M, Acosta N, Gaunt MW, Miles MA (2011). Recent, independent and anthropogenic origins of *Trypanosoma cruzi* hybrids. PLoS Negl Trop Dis.

[71] Monteiro WM, Teston APM, Gruendling AP, dos Reis D, Gomes ML, de Araújo SM (2013). *Trypanosoma cruzi* I and IV stocks from Brazilian Amazon are divergent in terms of biological and medical properties in mice. PLoS Negl Trop Dis.

[72] Sánchez-Moreno M, Sanz AM, Gómez-Contreras F, Navarro P, Marín C, Ramírez-Macias I (2011). In vivo trypanosomicidal activity of imidazole- or pyrazole-based benzo[g]phthalazine derivatives against acute and chronic phases of Chagas disease. J Med Chem.

[73] Sánchez-Moreno M, Gómez-Contreras F, Navarro P, Marín C, Olmo F, Yunta MJR (2012). Phthalazine derivatives containing imidazole rings behave as Fe-SOD inhibitors and show remarkable anti-*T. cruzi* activity in immunodeficient-mouse mode of infection. J Med Chem.

[74] Toledo MJO, Bahia MT, Cláudia MC, Martins-Filho OA, Tibayrenc M, Barnabe C (2003). Chemotherapy with benznidazole and itraconazole for mice infected with different *Trypanosoma cruzi* clonal genotypes. Antimicrob Agents Chemother.

[75] Phan IQH, Davies DR, Moretti NS, Shanmugam D, Cestari I, Anupama A (2015). Iron superoxide dismutases in eukaryotic pathogens: new insights from apicomplexa and *Trypanosoma* structures. Acta Crystallogr F Struct Biol Commun.

[76] Bachega JFR, Navarro MVAS, Bleicher L, Bortoleto-Bugs RK, Dive D, Hoffmann P (2009). Systematic structural studies of iron superoxide dismutases from human parasites and a statistical coupling analysis of metal binding specificity. Proteins.

