# Supplementary material for: Trypanosoma cruzi iron superoxide dismutases: insights from phylogenetics to chemotherapeutic target assessment
Source: Parasit Vectors. 2022 Jun 6;15:194. doi: 10.1186/s13071-022-05319-2 (PMC9169349; doi:10.1186/s13071-022-05319-2)
Supplement: Supplementary file 3 — Additional file 3: Table S1. Primers to amplify T. cruzi FeSOD-A and FeSOD-B genes. Abbreviations: Temp, Predicted annealing temperature. Figure S3. PCR amplification products of FeSOD-A and FeSOD-B genes of 8 T. cruzi strains. Electrophoresis was performed in a 1% agarose gel stained with GelRed™ (Biotium). The molecular weight markers 1-kb Plus DNA Ladder (Invitrogen) were used. Abbreviations: NC, Negative control of reaction without template. Table S2. Data of read depth and coverage analysis. The depth read values and coverage proportion in the genomic regions (GenBank: MZ825448–MZ825457) for each FeSOD type of genes and the respective normalized value (gene/genome depth). Depth was calculated using the value for each position of the gene. The coverage corresponds to the proportion of position that has reads in the gene, and normalized values are the depth of the genes divided by the mean of genome depth. Table S3. Sequence assembly information. Reads refers to the number of reads used for sequence assembly; Consensus refers to the length (nt) of the consensus sequence. Sequence assembly was performed using Phred-Phrap-Consed. [file 13071_2022_5319_MOESM3_ESM.docx]

**Table S1.** Primers to amplify *T. cruzi* FeSOD-A and FeSOD-B genes

| Gene | Direction | Primer sequence (5’-3’) | GC (%) | Temp.  (Celsius) | Primer Length  (mer) |
| --- | --- | --- | --- | --- | --- |
| FeSOD-A | Forward | ATGTTGAGACGTGCGGTGAATATATC | 42.3 | 56.4 | 26 |
|  | Reverse | TCAAGTAATCCGGCCGGCGGTTTTC | 56 | 61 | 25 |
| FeSOD-B | Forward | ATGGTCTTCAGCATTCCTCCGC | 54.5 | 56.7 | 22 |
|  | Reverse | GTTCCACGTTCTTCCAGTTGAC | 50 | 54.8 | 22 |
| FeSOD-A  (internal) | Forward | CCCTTGAGTCTGCTGTTACGGCC | 60.8 | 61 | 23 |
|  | Reverse | TCCAACCCGAGCCAAAGTTATT | 45.4 | 53 | 22 |
| FeSOD-B  (internal) | Forward | TTAACCACACGTTCTACTGGG | 47.6 | 52 | 21 |
|  | Reverse | CAAGCCACGCCCAACCCGAG | 70 | 60 | 20 |

Temp: predicted annealing temperature

**
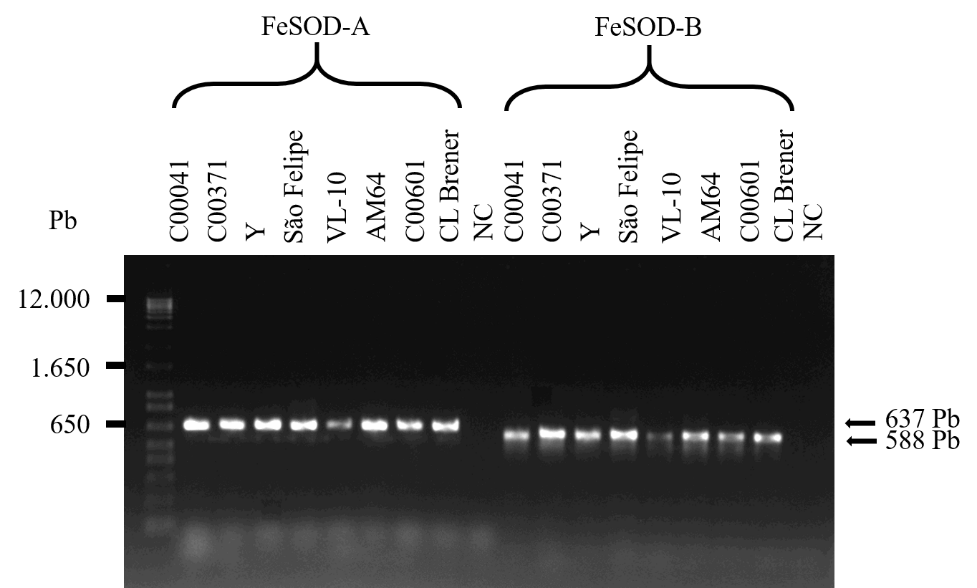
**

**Figure S3.** PCR amplification products of FeSOD-A and FeSOD-B genes of eight *T. cruzi* strains. Electrophoresis was performed on a 1% agarose gel stained with GelRed™ (Biotium). NC: negative control of reaction without template. The molecular weight markers 1 Kb Plus DNA Ladder (Invitrogen) were used

**Table S2.** Data of read depth and coverage analysis

| Isoform | Scaffold | Start  position | End  position | Coverage | Mean depth | Normalized depth |
| --- | --- | --- | --- | --- | --- | --- |
| FeSOD-A | TcBrS006 | 610002 | 610703 | 100 | 68 | 1.48 |
|  | TcBrS020 | 649023 | 649724 | 100 | 45 | 0.98 |
| FeSOD-B | TcBrS024 | 375771 | 376394 | 100 | 43 | 0.93 |
|  | TcBrS074 | 371524 | 372111 | 100 | 51 | 1.11 |
|  | TcBrS110 | 279843 | 280466 | 100 | 46 | 1.00 |
|  | TcBrS188 | 30050 | 30637 | 100 | 42 | 0.91 |
| FeSOD-C | TcBrS091 | 215034 | 216071 | 100 | 44 | 0.96 |
|  | TcBrS091 | 219697 | 220734 | 100 | 50 | 1.09 |
|  | TcBrS112 | 47508 | 48542 | 100 | 41 | 0.89 |
|  | TcBrS112 | 52180 | 53214 | 100 | 40 | 0.87 |

The depth read values and coverage proportion in the genomic regions (GenBank access: MZ825448-MZ825457) for each FeSOD type of genes and the respective normalized value (gene/genome depth). Depth was calculated using the value for each position of the gene. Already the coverage corresponds to the proportion of position that has reads in the gene and normalized values are the depth of the genes divided by the mean of genome depth

**Table S3.** Sequence assembly information

| *T. cruzi* | FeSOD-A gene | | FeSOD-B gene | |
| --- | --- | --- | --- | --- |
| Strains/Clones | Reads | Consensus | Reads | Consensus |
| 2137 | 14 | 625 | 14 | 563 |
| 2549 | 14 | 625 | 14 | 574 |
| 3253 | 14 | 615 | 13 | 524 |
| AM64 | 12 | 612 | 14 | 561 |
| Berenice | 14 | 610 | 14 | 528 |
| Bug2149cl10 | 14 | 608 | 14 | 530 |
| Buriti | 14 | 612 | 14 | 539 |
| C00041 | 14 | 610 | 14 | 560 |
| C00113 | 12 | 608 | 14 | 534 |
| C00370 | 14 | 636 | 14 | 538 |
| C00471 | 14 | 610 | 14 | 563 |
| C00524 | 13 | 634 | 14 | 518 |
| C00526 | 14 | 636 | 14 | 558 |
| C00566 | 14 | 636 | 12 | 562 |
| C00601 | 14 | 609 | 14 | 563 |
| CL | 14 | 636 | 14 | 558 |
| CL_Brener | 14 | 611 | 14 | 534 |
| Colombiana | 14 | 609 | 14 | 537 |
| Ernane | 14 | 613 | 14 | 529 |
| FL | 14 | 630 | 14 | 573 |
| Gilmar | 13 | 623 | 14 | 534 |
| JM | 14 | 636 | 14 | 537 |
| Luna | 14 | 635 | 14 | 534 |
| MR | 13 | 623 | 14 | 547 |
| PEBA18 | 14 | 624 | 14 | 531 |
| PL0213 | 14 | 634 | 14 | 560 |
| Quaraizinho | 14 | 611 | 14 | 552 |
| RN19 | 14 | 636 | 14 | 515 |
| RS-12 | 14 | 633 | 14 | 559 |
| RS-21 | 14 | 634 | 14 | 526 |
| São Felipe | 14 | 595 | 14 | 531 |
| SC28 | 14 | 611 | 14 | 562 |
| SM76 | 14 | 634 | 14 | 557 |
| VL-10 | 7 | 589 | 10 | 565 |
| Y | 14 | 610 | 14 | 530 |

Reads: number of reads used for sequence assembly. Consensus: length (nt) of the consensus sequence. Sequence assembly was performed using Phred-Phrap-Consed
