# Supplementary material for: Trypanosoma cruzi iron superoxide dismutases: insights from phylogenetics to chemotherapeutic target assessment
Source: Parasit Vectors. 2022 Jun 6;15:194. doi: 10.1186/s13071-022-05319-2 (PMC9169349; doi:10.1186/s13071-022-05319-2)
Supplement: Supplementary file 8 — Additional file 8: Table S5.In vitro trypanocidal activity, cytotoxicity and selectivity index of selected drugs. Superscripts: 1Reduction (%) of the amastigote and trypomastigote by drug; 2drug concentration that inhibits 50% of the growth of the amastigotes and trypomastigotes of T. cruz;3drug concentration that inhibits 50% of the L929 cell viability;4CC50 L929/IC50 T. cruz. IC50 and CC50 values were calculated by linear interpolation. The determination of CC50 of polaprezinc was not possible due low solubility in concentrations > 50 μM. Abbreviations: ND, Not determined. Fig. S7. The diagram illustrates FeSOD-A, FeSOD-B and FeSOD-C characteristics, including cellular localization (orange boxes), protein length (blue boxes), gene copies (green boxes) and protein domains (pink boxes). Information was retrieved from public databases and tools: ENA, Pfam, TriTrypDB, and UniProt. Reference sequences were selected for FeSOD-A (AAC47548.2), FeSOD-B (EAN92179.1) and FeSOD-C (TcCLB.511735.60). Only gene copy numbers were obtained by our prediction using the T. cruzi CL-Brener genome. [file 13071_2022_5319_MOESM8_ESM.docx]

**Table S5** *In vitro* trypanocidal activity, cytotoxicity, and selectivity index of selected drugs

| Compound | Concentration  (μM) | Activity^1^  (%) | IC_50_ *T. cruzi*^2^  (μM) | CC_50_ L929^3^  (μM) | Selectivity index (SI)^4^ |
| --- | --- | --- | --- | --- | --- |
| Polaprezinc | 1,000  500  250  125  62.5 | Cell death  Cell death  Cell death  Cell death  14 | Inactive | ND | ND |
| Mangafodipir | 1,000  500 | 69  10 | 839 | 2,298 | 2.7 |
| Benznidazole | 3.8 | 83 | 3.81  (1 μg/mL) | 2,381  (625 μg/mL) | 625 |

^1^ Reduction (%) of the amastigote and trypomastigote by drug; ^2^Drug concentration that inhibits 50% of the growth of the amastigotes and trypomastigotes of *T. cruzi*; ^3^Drug concentration that inhibits 50% of the L929 cell viability; ^4^CC_50_ L929/IC_50_ *T. cruzi*. IC_50_ and CC_50_ values were calculated by linear interpolation. ND-not determined: The determination of CC_50_ of polaprezinc was not possible due low solubility in concentrations > 50 μM


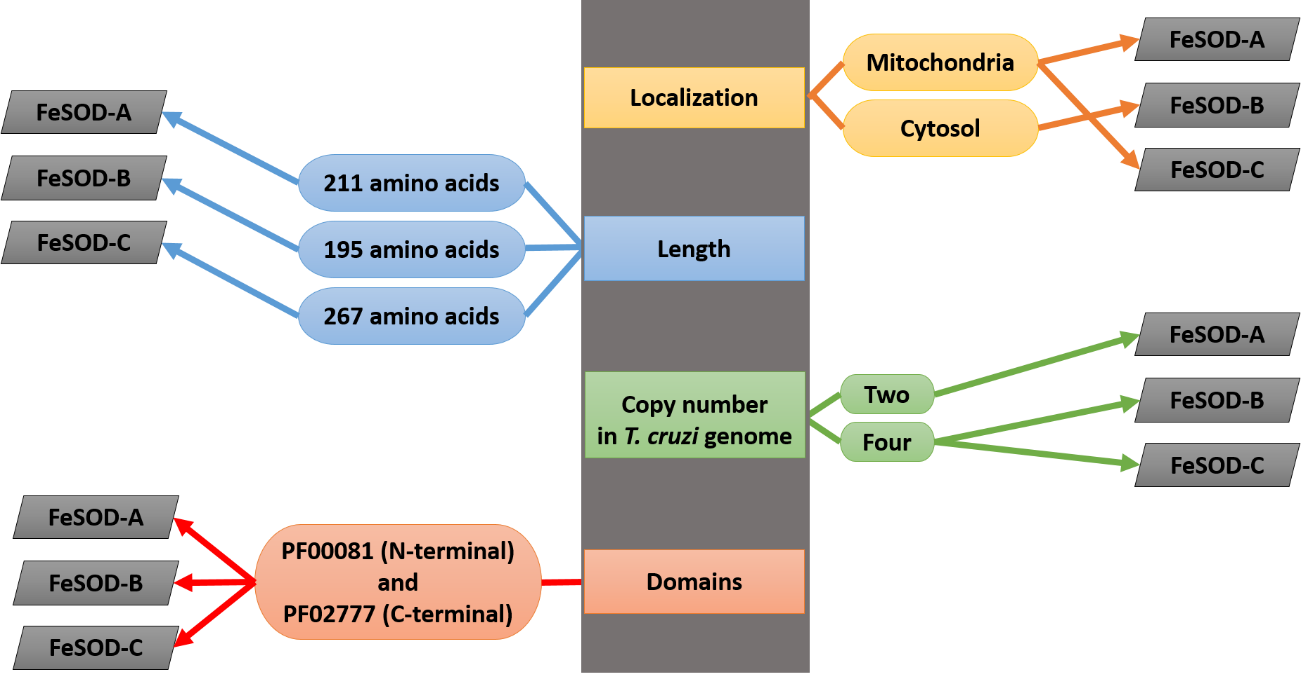


**Figure S7.** The diagram illustrates FeSOD-A, FeSOD-B, and FeSOD-C characteristics. They include: cellular localization (orange boxes), protein length (blue boxes), gene copies (green boxes) and protein domains (pink boxes). Information was retrieved from public databases and tools: ENA, Pfam, TriTrypDB, and UniProt. Reference sequences were selected for FeSOD-A (AAC47548.2), FeSOD-B (EAN92179.1), and FeSOD-C (TcCLB.511735.60). Only gene copy numbers were obtained by our prediction using the *T. cruzi* CL Brener genome
